# Microwave-induced electric discharges on metal particles for the synthesis of inorganic nanomaterials under solvent-free conditions

**DOI:** 10.3762/bjnano.11.86

**Published:** 2020-07-13

**Authors:** Vijay Tripathi, Harit Kumar, Anubhav Agarwal, Leela S Panchakarla

**Affiliations:** 1 Department of Chemistry, Indian Institute of Technology Bombay, Powai, Mumbai 400076, India

**Keywords:** electric discharges, microwave synthesis, nanomaterials, transmission electron microscopy

## Abstract

Microwave irradiation of metals generates electric discharges (arcs). These arcs are used to generate nanoparticles of Cu and Ni and one-dimensional nanorods of CuS, ZnF_2_, and NiF_2_ protected with fluorinated amorphous carbon. We have also synthesized reduced graphene oxide and partially rolled graphene by this method.

## Introduction

The synthesis of nanomaterials in short time intervals with fewer chemicals has become increasingly important in materials science. Traditional routes of synthesizing nanomaterials, including sol–gel synthesis, solvothermal synthesis, arc-discharge synthesis, or laser ablation, require either large amounts of chemicals or longer synthesis times, or both [1]. Microwave synthesis has become popular in the last three decades as an alternative route for synthesizing molecules and materials at a significantly shorter time scale [2–8]. Dielectric heating under microwave irradiation both in solution and in the solid state rapidly increases the reaction temperature and helps to improve reaction kinetics significantly. This reduces drastically the reaction time [9–10]. Non-thermal effects may also influence the reaction kinetics, which is still a subject of discussion [9,11]. Bulk metals generally reflect microwaves, whereas fine metal powders or thin films can couple with microwaves (the penetration depth of microwaves in metals is 1–2 µm). This will quickly increase the temperature through conduction mechanisms, which enables the sintering of metals by using microwaves [12–14]. It was found that the sintering of metal powders by microwaves produces products that are denser than and mechanically superior to the ones obtained by conventional heating [14]. Other than reflection and conduction, metal particles usually produce electric discharges (arcs) when exposed to microwaves due to the formation of high electric field gradients at sharp edges on the metal surfaces [12]. The generation of arcs might be the reason why microwave irradiation has not been used to generate different nanomaterials from metal particles. However, some studies show that the treatment of metals under microwave irradiation in organic solvents can carbonize the organic solvents forming carbon-coated metallic nanoparticles [15–16]. Recently, Pentsak et al. have shown that metals, such as Cu, Fe, and Mo, on carbon form nanometer-scale structures under microwave heating [17]. However, the microwave discharge technique, which is fast, solvent-free, and easy to set up technically, has not been explored to its fullest potential to synthesize different nanomaterials with controlled morphology.

In this communication, we report on the microwave-induced electric discharge synthesis of Cu, Ni, und Zn nanoparticles from metal particles. Also, we can control the morphology of the nanomaterials, which has not been achieved before. ZnF_2_, NiF_2_, and CuS nanorods covered with amorphous fluorinated carbon were synthesized. We have also extended this procedure to synthesize reduced graphene oxide and graphene without using any solvents or additional surfactants.

## Results and Discussion

**Figure 1:**
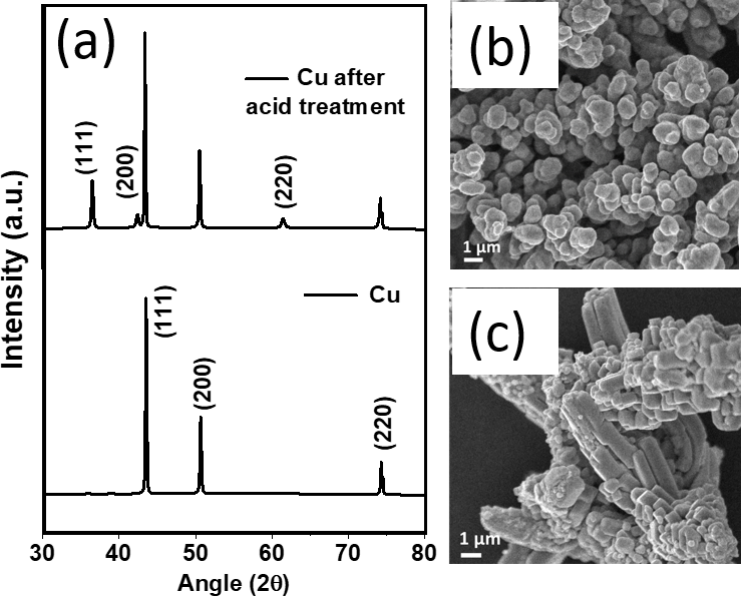
(a) XRD patterns of commercially available Cu powder and Cu powder after treatment with 0.5 M HNO_3_. (b) SEM images of pure Cu powder and (c) acid-treated Cu powder.

Smooth surfaces on commercially available metal particles do not create arcs under microwave irradiation. Instead, they heat up or reflects the microwaves. Thus, activating metal surfaces by acid treatment is essential before using metal particles in further microwave arc experiments. All metal powders were treated with 0.5 M nitric acid under sonication for 10 min to create rough surfaces. In this process, copper partially gets oxidized to Cu_2_O. The X-ray diffraction (XRD) patterns show reflections of Cu_2_O after acid treatment, while the majority of Cu remains in the metallic form (Figure 1a). Scanning electron microscopy (SEM) images of copper powder before and after acid treatment are shown in Figure 1b and Figure 1c, respectively. The SEM image clearly shows the sharp edges on acid-treated copper. Graphitic carbon nitride (g-C_3_N_4_) or graphite powder (commercially available) are used as carbon source. g-C_3_N_4_ is synthesized and characterized according to [18]. X-ray diffraction (XRD) and X-ray photoelectron spectroscopy (XPS) confirms the formation of g-C_3_N_4_ (Figure S1 in Supporting Information File 1).

The reactions were carried out in quartz or Teflon beakers. A Teflon beaker also served as a carbon and fluorine source for the experiments. Typically, 100 mg of acid-treated metal powder mixed either with graphite powder or g-C_3_N_4_ (50 mg) were placed inside a domestic kitchen microwave (2.54 GHz, power 700 W) and treated for periods of time between 5 s and 2 min. Arcs were generated in the activated metal, which evaporated the metal along with carbon. Carbon-coated metallic nanoparticles formed on the top lid of the reaction vessel. For synthesizing nanorods, sulfur was used as a growth promoter. Typically, a mixture of activated metal (100 mg), sulfur powder (25 mg) and g-C_3_N_4_ (50 mg) were added to a Teflon beaker and irradiated with microwaves. It is important to note that in the absence of carbon (graphite/g-C_3_N_4_), the arc synthesis yielded a mixture of metal and metal oxide nanoparticles and the particle sizes were found to be difficult to control.

**Figure 2:**
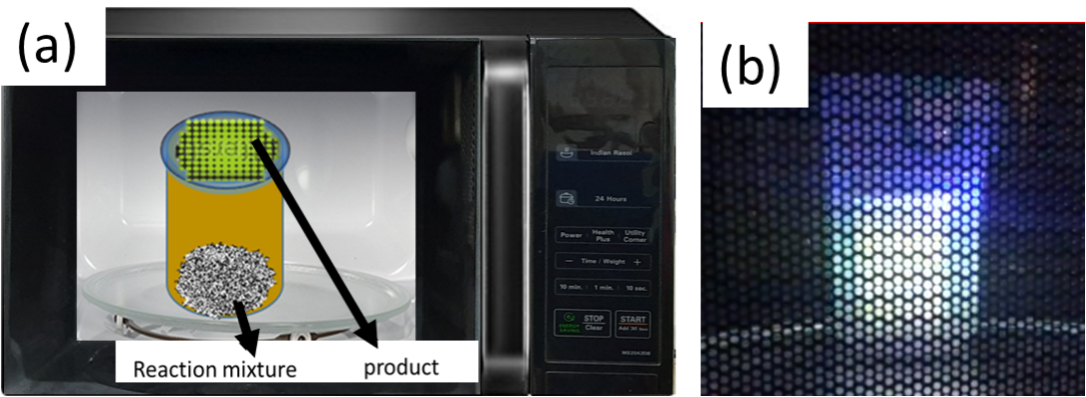
(a) Schematic of the synthesis in a microwave reactor, (b) plasma generated with metal particles under microwave irradiation.

**Figure 3:**
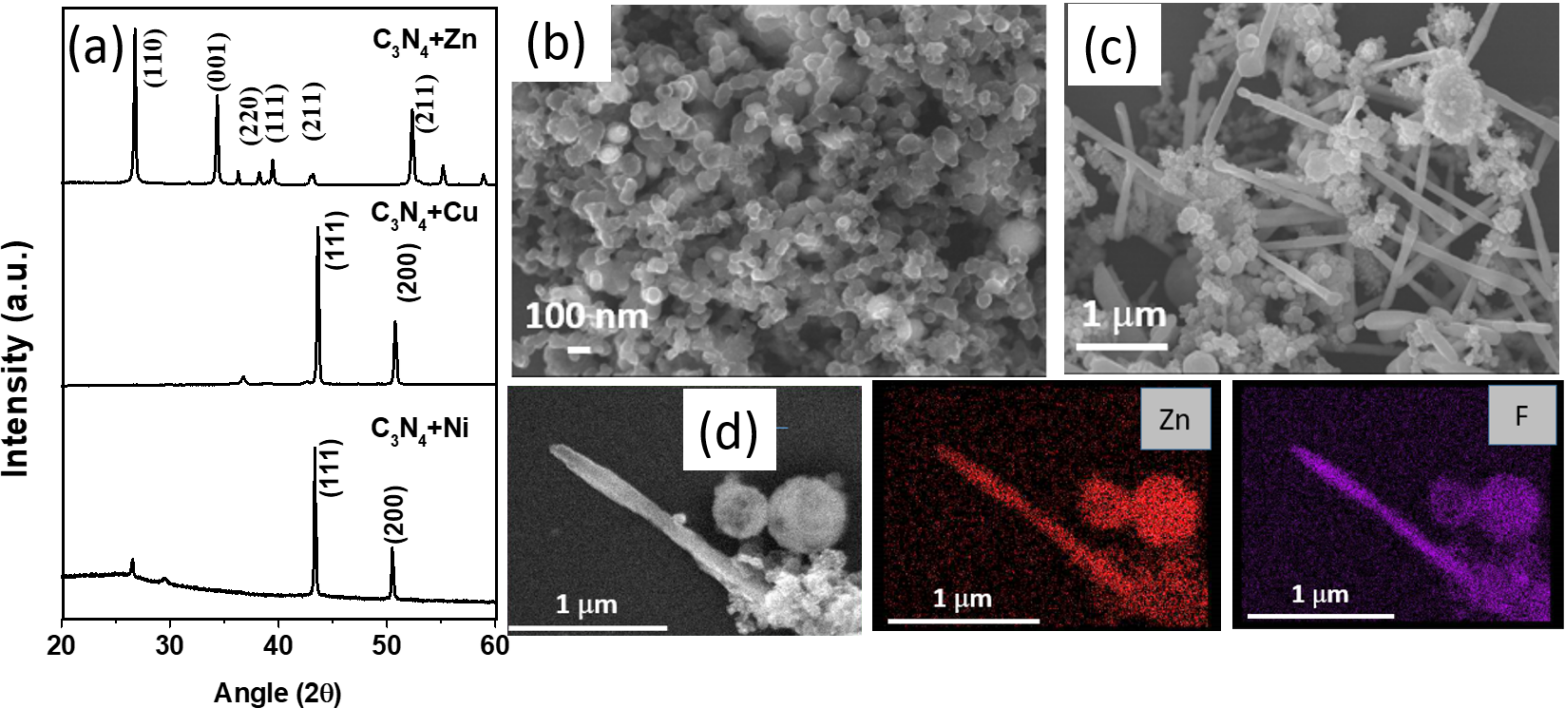
(a) XRD patterns of carbon-coated Cu and Ni nanoparticles and ZnF_2_ nanorods. (b) SEM images of carbon-coated Cu nanoparticles. (c) SEM images of ZnF_2_ nanorods. (d) SEM image and EDS elemental mapping of a single ZnF_2_ nanorod.

In Figure 2a, we show a schematic of a reaction vessel inside a microwave device. Figure 2b shows the optical image of plasma generated in the reaction vessel during microwave irradiation. Microwave irradiation of activated metals mixed with either graphite or graphitic carbon nitride (g-C_3_N_4_) yields carbon-coated or nitrogen-doped carbon-coated metallic nanoparticles. When these reactions are conducted in a Teflon reactor, the products are further functionalized with fluorine. Figure 3a shows XRD patterns of Cu and Ni nanoparticles generated after 1 min of microwave treatment of activated Cu and Ni powders in the presence of g-C_3_N_4_. The patterns show pure phases of Ni and Cu. The formed nanoparticles are covered with fluorinated amorphous carbon. Figure 3b shows the SEM image of Cu nanoparticles covered with amorphous fluorinated carbon with an average size of 80 nm. The C–F bonds at the surface of the metallic nanoparticles could be further functionalized for different applications such as drug delivery [19]. A SEM image of Ni nanoparticles is shown in Figure S2 in Supporting Information File 1. Figure S3 in Supporting Information File 1 shows the SEM images of Cu nanoparticles covered with amorphous fluorinated carbon synthesized using graphite as carbon source. The structural and morphological features of these Cu nanoparticles are similar to those of Cu nanoparticles produced using g-C_3_N_4_. In contrast to Cu and Ni, microwave irradiation of zinc metal in the presence of Teflon and g-C_3_N_4_ creates ZnF_2_ nanorods inside fluorinated carbon. From XPS (Figure S4, Supporting Information File 1) the carbon-to-fluorine ratio was calculated to be 3:2. Zn is highly electropositive and reacts readily with fluorine from Teflon and forms ZnF_2_. The XRD patterns in Figure 3a confirm the formation of ZnF_2_. Both nanorods and nanoparticles of ZnF_2_ were formed as can be seen from the SEM image (Figure 3c). Figure 3d shows the energy-dispersive spectroscopy (EDS) mapping of Zn and F, which confirms the presence of Zn and F in the nanorod. The average diameter of the nanorods is 100 nm and the length ranges from 2 to 3 µm. The formation of external amorphous fluorinated carbon nanotubes helps as a template in the formation of ZnF_2_ nanorods.

It is well established that sulfur acts as a growth promoter for carbon nanotubes and carbon fibers [20–21]. Thus, sulfur was introduced to the reaction mixtures to improve the yield of nanorods. The microwave treatment of activated Zn metal with g-C_3_N_4_ and sulfur in a Teflon container produced ZnF_2_ nanorods in high yield. The XRD patterns in Figure S5 in Supporting Information File 1 confirm the phase purity of ZnF_2_. Figure S6 in Supporting Information File 1 shows the SEM and transmission electron microscopy (TEM) images of ZnF_2_ nanorods produced in the presence of sulfur. The SEM images indicate the high yield of ZnF_2_ nanorods. The high-resolution TEM (HRTEM) image in Figure S6d (Supporting Information File 1) confirms the single-crystalline nature of ZnF_2_. Similarly, microwave treatment of Ni in the presence of sulfur in a Teflon beaker yielded NiF_2_ nanorods along with Ni nanoparticles (Figure S5 and Figure S7, Supporting Information File 1). It is important to note that a similar experiment without sulfur yielded only Ni nanoparticles. Figure S7 in Supporting Information File 1 shows SEM and TEM images of NiF_2_ nanorods. The EDS mapping in Figure S7c (Supporting Information File 1) confirms the presence of Ni, F and C in the NiF_2_ nanorods. A TEM image of NiF_2_ nanorod covered with amorphous carbon is shown in Figure S7d (Supporting Information File 1). The HRTEM image (Figure S7e, Supporting Information File 1) clearly shows the single-crystalline nature of the NiF_2_ nanorod.

**Figure 4:**
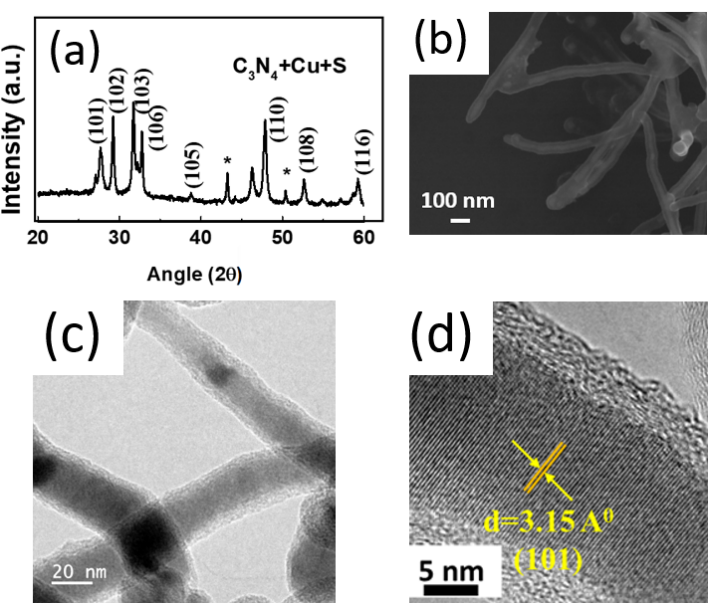
(a) XRD patterns of CuS nanorods synthesized in the presence of sulfur by microwave irradiation of copper in the presence of g-C_3_N_4_ in a Teflon beaker for 2 min. (b) SEM, (c) TEM and (d) HRTEM images of CuS nanorods. The asterisk (*) in panel (a) indicates the reflections from Cu.

Interestingly, microwave treatment of copper in the presence of sulfur in Teflon yielded CuS nanorods instead of CuF/CuF_2_ nanorods. The reactivity of Cu with sulfur is higher than that with fluorine as soft–soft interactions between Cu and S dominate the product stability compared to soft–hard interactions between Cu and F. The XRD pattern in Figure 4a confirms the hexagonal covellite structure of CuS. The SEM image in Figure 4b and the TEM image in Figure 4c confirm the one-dimensional nature. CuS nanorods are single-crystalline as can be seen from the HRTEM image in Figure 4d. CuS nanorods were found to grow along the [101] direction. The average core diameter of the CuS nanorods is about 25 nm and the thickness of the amorphous layer on top of the CuS nanorods is about 5 nm.

Reports on the generation of inorganic nanomaterials from metals by electric arcs under microwave irradiation are rare. Here, we have shown that metals can effectively interact with microwaves when metallic particles contain rough surfaces or sharp edges. When electrically conducting rough surfaces are exposed to microwaves, electric fields distribute inhomogeneously along the surface of the conductor. At tips and sharp edges, very high electric field gradients occur, leading to the ionization of the material and the surrounding gas, followed by electric discharges [12]. These discharges might lead to the melting of the metal and the evaporation of metal, surrounding carbon, sulfur, and fluorine (from Teflon). A chemical reaction between the metal and sulfur and/or fluorine leads to the formation of metal sulfides and/or fluorides depending on the reactivity. Cu and Ni, in the presence of graphite and /or g-C_3_N_4_ and the absence of sulfur produce metallic nanoparticles. This might be due to the reducing capability of carbon at high temperatures preventing the metals from getting oxidized. When sulfur is used in the reaction mixture along with g-C_3_N_4_, sulfur helps to produce carbon nanotubes instead of carbon nanospheres. The reactivity of sulfur with the end caps of nanotubes does not allow the carbon nanotubes to close. These amorphous carbon nanotubes help as a template to intercalate metal fluorides/metal sulfides. As the local temperatures are very high, metal fluorides and sulfides melt and fill the nanotubes via capillary forces. The liquids solidify as one-dimensional nanorods inside the nanotubes. The usage of g-C_3_N_4_, instead of graphite, was found to be beneficial, especially for obtaining high yields of nanorods. However, the choice of graphite or g-C_3_N_4_ has shown little to no difference in the synthesis of nanoparticles.

**Figure 5:**
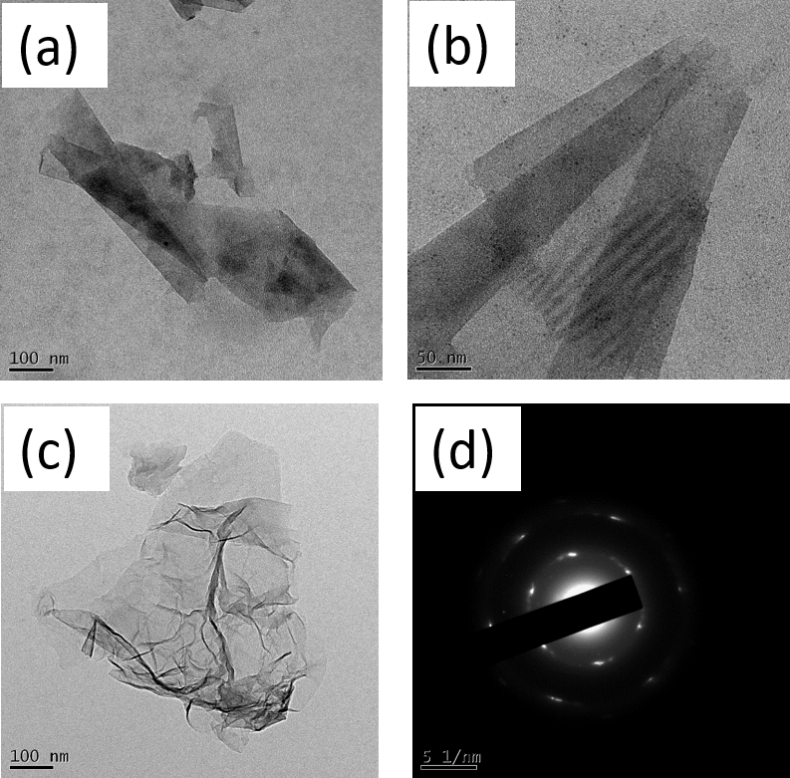
(a, b) TEM images of few-layered graphene partially rolled into nanoscrolls synthesized by irradiating graphite with microwaves in the presence of Zn metal for 30 s. (c) TEM image of graphite oxide after exfoliation trough microwave irradiation in the presence of Zn metal for 30 s and (d) the corresponding selected area electron diffraction pattern.

We have also studied exfoliation of graphite and graphite oxide (GO) under microwave irradiation in the presence of Zn metal. We have observed exfoliation of graphite into few-layered graphene. We have also seen a partial rolling of graphene sheets into nanoscrolls (Figure 5a,b). The formation of ultrasmall nanoparticles of ZnO along with graphene was detected (see Figure S8 in Supporting Information File 1). In the case of GO exfoliation, we have observed the formation of nanosheets of reduced graphite oxide (Figure 5c). These nanosheets still keep hexagonal structure under microwave irradiation as can be seen from the selected area electron diffraction pattern in Figure 5d.

## Conclusion

We have shown that microwave-induced electric discharges on rough metallic surfaces can be effectively used to synthesize nanomaterials with controlled morphology. Cu and Ni metallic nanoparticles are stabilized through amorphous carbon. Nanorods of ZnF_2_, NiF_2_ and CuS are synthesized inside fluorinated amorphous carbon nanotubes in the presence of sulfur. External C–F bonds can be further functionalized readily without disturbing internal materials. We could also produce reduced graphene oxide and graphene partially rolled into nanoscrolls. We hope that this work encourages further research exploring the possibilities to synthesize other inorganic nanomaterials by microwave-induced electric discharge without the need for surfactants and solvents.

## Experimental

### Roughening the surface metal powders

Commercially purchased micrometer-sized metal powders (Ni, Cu and Zn) are treated with nitric acid to create rough surfaces. In a typical reaction, 100 mg of metal powder is transferred to a beaker containing 10 mL of 0.5 M nitric acid and sonicated for 10 min. The resultant powder is washed with water several times until pH 7 and dried it in an oven at 50 °C for 2 h before being used in further microwave experiments.

### Synthesis of graphitic carbon nitride (g-C_3_N_4_)

g-C_3_N_4_ is synthesized and characterized according to [18]. In a typical reaction, melamine (150 mg) and urea (71 mg) are mixed in a quartz boat and heated at 650 °C under nitrogen flow for 2 h to obtain bulk g-C_3_N_4_ as orange product.

### Generation of nanomaterials using microwave-induced discharge

To generate nanoparticles by microwave-induced discharge, the reaction is conducted either in a quartz or a Teflon beaker. A Teflon beaker also serves as a carbon and fluorine source for the experiments. Typically, 100 mg of acid-treated metal powder mixed either with graphite powder or g-C_3_N_4_ (50 mg) and placed inside a domestic kitchen microwave (2.54 GHz, power 700 W) and treated for 5 s to 2 min. The product containing carbon-coated metallic nanoparticles are collected from the lid of the reaction vessel. For synthesizing nanorods, sulfur is used as a growth promoter. Typically, a mixture of activated metal (100 mg), sulfur powder (25 mg) and g-C_3_N_4_ (50 mg) is placed in a Teflon beaker and irradiated with microwaves. The products are collected from the lid of the reaction vessel. The above experiments are performed in air.

### Preparation of few-layer graphene and graphite oxide nanosheets

In a typical experiment, 50 mg of acid-treated Zn metal is mixed with 100 mg of either graphite or graphite oxide in a quartz beaker and irradiated with microwaves for 1 min. Products are collected from the reaction vessel, sonicated in ethanol for 1 min and centrifuged for 5 min at 6000 rpm to separate big metal particles and unreacted graphite. The supernatant solution is collected for further characterization.

### Characterization of products

The reaction products are characterized by X-ray diffraction (PANanalytical X’pert PRO), field-emission scanning electron microscopy (JEOL JSM 7600F, combined with energy-dispersive spectroscopy (EDS)), transmission electron microscopy (JEOL JEM 2100F) and X-ray photoelectron spectroscopy XPS (Thermo VG Scientific MultiLab, ESCA).

## Supporting Information

File 1 Characterization details of g-C_3_N_4_ by XRD and XPS. Electron microscope analysis of Ni, Cu, ZnF_2_, NiF_2_, and ZnO nanostructures.
