# Supplementary material for: Microwave-induced electric discharges on metal particles for the synthesis of inorganic nanomaterials under solvent-free conditions
Source: Beilstein J Nanotechnol. 2020 Jul 13;11:1019–25. doi: 10.3762/bjnano.11.86 (PMC7372228; doi:10.3762/bjnano.11.86)
Supplement: File 1 — Characterization details of g-C3N4 by XRD and XPS. Electron microscope analysis of Ni, Cu, ZnF2, NiF2, and ZnO nanostructures. [file Beilstein_J_Nanotechnol-11-1019-s001.pdf]

BEILSTEIN JOURNAL OF NANOTECHNOLOGY

Supporting Information

for

Microwave-induced electric discharges on metal particles for the synthesis of inorganic nanomaterials under solvent-free conditions

Vijay Tripathi, Harit Kumar, Anubhav Agarwal and Leela S. Panchakarla

Beilstein J. Nanotechnol. **2020,** *11,* 1019–1025. doi:10.3762/bjnano.11.86

Characterization details of g-C_3N_4 by XRD and XPS. Electron microscope analysis of Ni, Cu, ZnF_2, NiF_2, and ZnO nanostructures

License and Terms: This is a supporting information file under the terms of the Creative Commons Attribution License (http://creativecommons.org/licenses/by/4.0). Please note that the reuse, redistribution and reproduction in particular requires that the authors and source are credited.
The license is subject to the *Beilstein Journal of Nanotechnology* terms and conditions: (https://www.beilstein-journals.org/bjnano)

Thermal decomposition of physically mixed melamine with urea in a tube furnace at 600 °C for 4 h in argon atmosphere yielded orange precipitates of graphitic carbon nitride (g-C_3N_4). The XRD pattern (Figure S1a) and the XPS spectrum (Figure S2b) match with reported literature [1], which confirms the formation of g-C_3N_4.

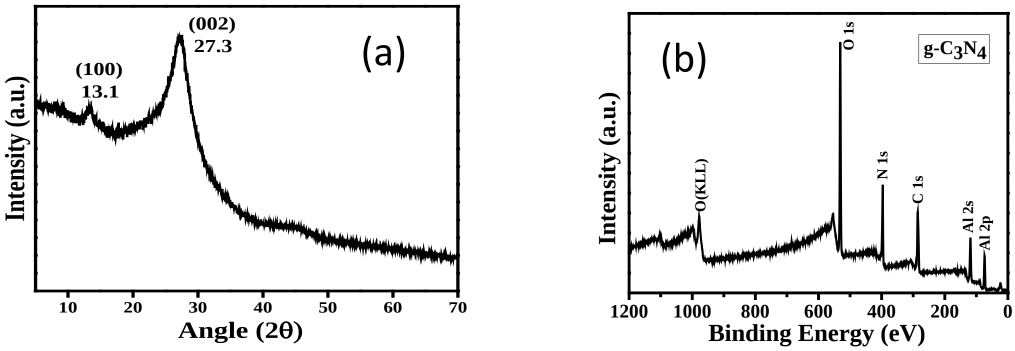

Figure S1: (a) XRD patterns and (b) XPS of as prepared g-C_3N_4.

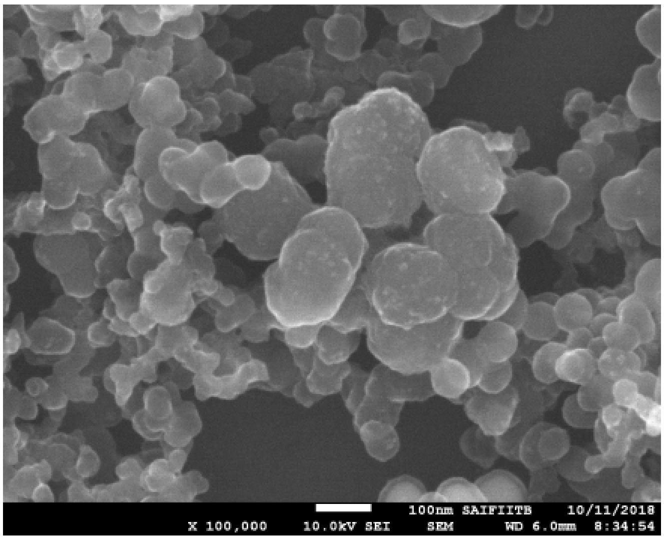

Figure S2: SEM image of Ni nanoparticles covered with amorphous carbon prepared by irradiating Ni metal with microwaves in the presence of g-C_3N_4 in a Teflon beaker for 1 min.

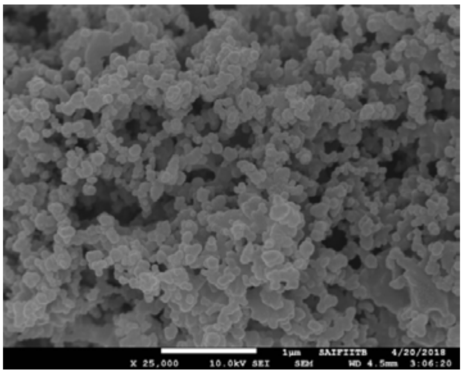

Figure S3: SEM image of Cu nanoparticles prepared by irradiation of Cu metal with microwaves in the presence of graphite in a Teflon beaker for 1 min.

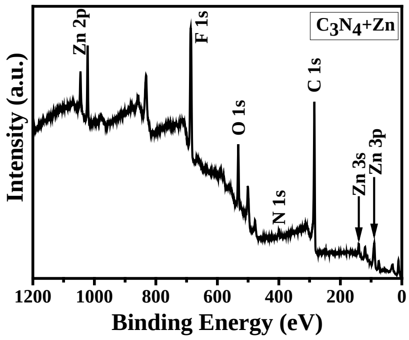

Figure S4: XPS patterns of ZnF_2 nanorods prepared by irradiating zinc metal with microwaves in the presence of g-C_3N_4 in a Teflon beaker for 1 min.

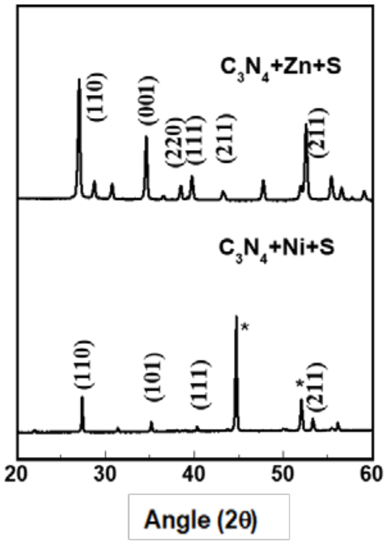

Figure S5: XRD patterns of nanorods of ZnF_2 and NiF_2 synthesized in the presence of sulfur by microwave irradiation in the presence of g-C_3N_4 in a Teflon beaker for 2 min.

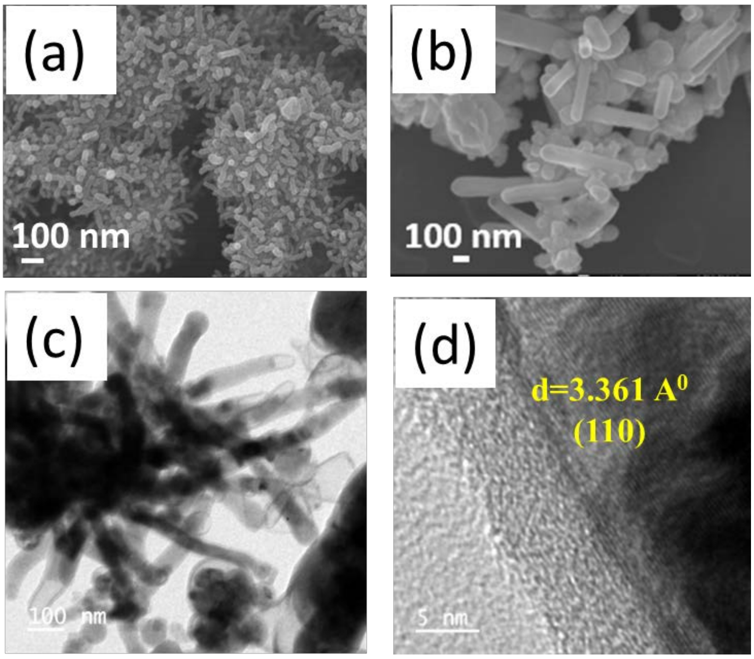

Figure S6: (a, b) SEM, (c) TEM, and (d) HRTEM images of ZnF_2 nanorods synthesized by microwave irradiation of Zn with sulfur and g-C_3N_4 in a Teflon beaker for 2 min.

Microwave irradiation of Ni with sulfur and g-C_3N_4 in a Teflon beaker yielded NiF_2 nanorods. The formation of NiF_2 nanorods along with Ni nanoparticles covered with fluorinated amorphous carbon can be seen in SEM images (Figure S7a,b). The SEM image and the EDS mapping of Ni, F and C on top of one of the NiF_2 nanorods (Figure S7c) confirms the presence of Ni, F and C. The TEM image in Figure S7d and the inset within confirm the amorphous coating on the NiF_2 nanorod. The HRTEM image in Figure S7e confirms the single-crystalline nature of the nanorods with <111> growth direction.

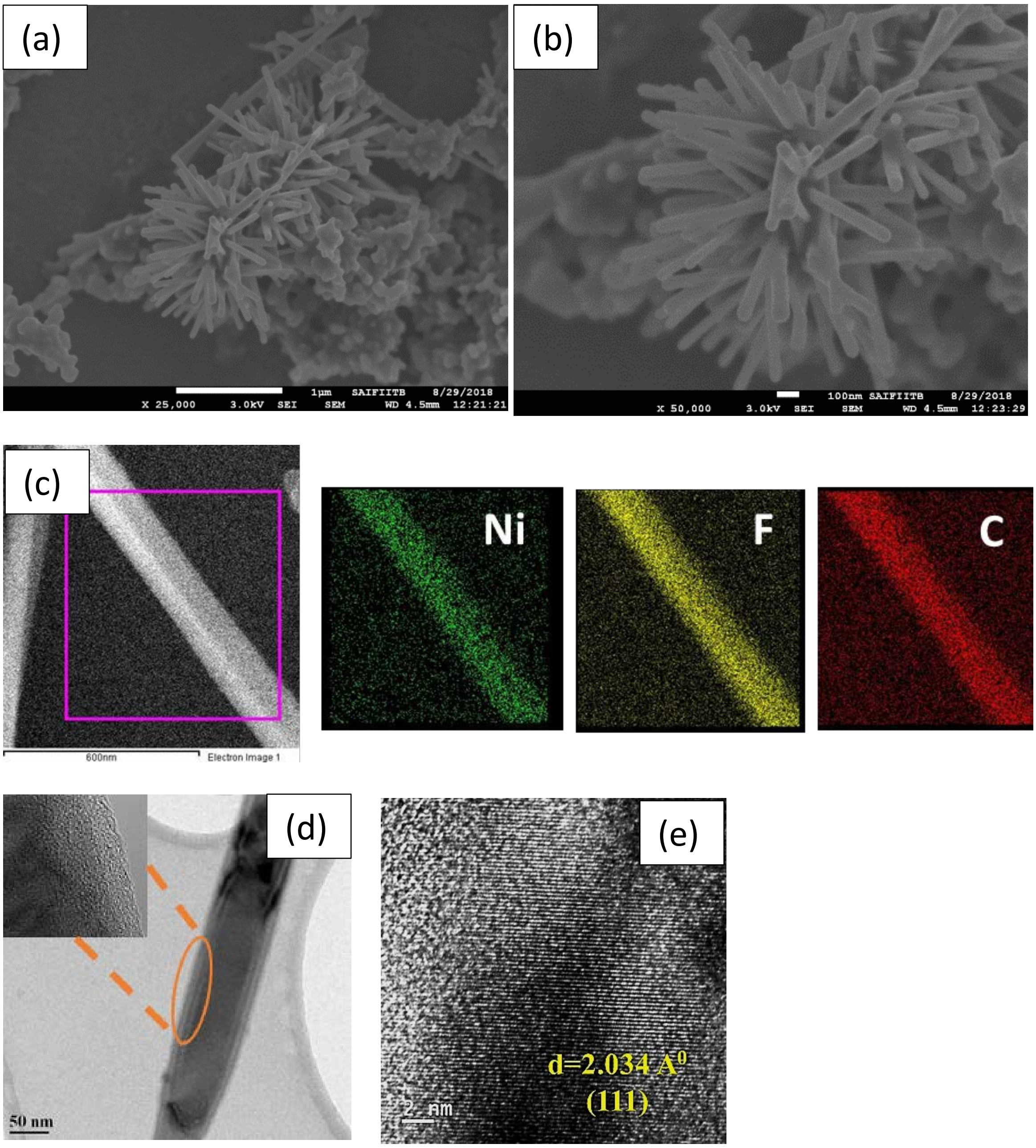

Figure S7: (a, b) SEM images and (c) EDS elemental mapping of Ni, F, C on one of the NiF_2 nanorods synthesized by microwave irradiation of Ni with sulfur and g-C_3N_4 in a Teflon beaker for 2 min. (d) TEM and (e) HRTEM image of the NiF_2 nanorod. The inset in (d) shows the magnified image at the edge of the nanorod.

Exfoliation of graphite in the presence of Zn metal under microwave irradiation produced few-layered graphene along with nanoparticles of ZnO. A HRTEM image of ZnO nanoparticle is shown in Figure S8. The interlayer lattice spacing of 0.282 nm is attributed to the (100) planes of ZnO.

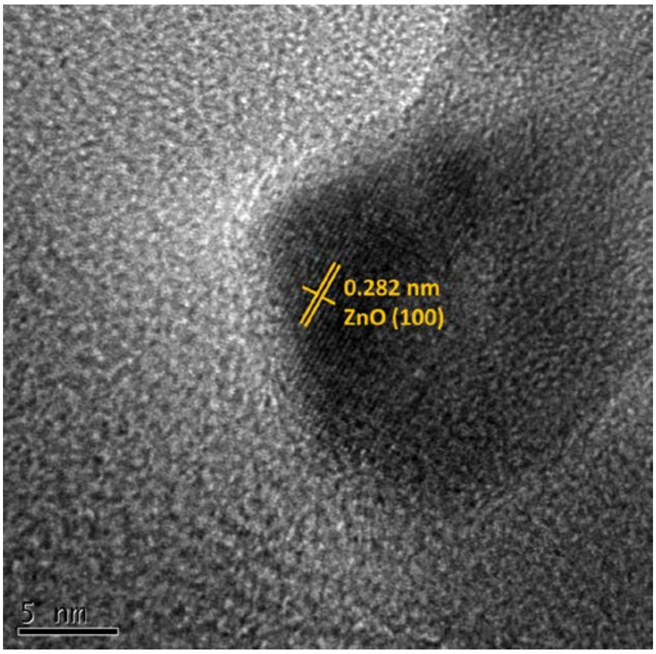

Figure S8: HRTEM image of ZnO nanoparticle

References

1. Dante, R. C.; Martín-Ramos, P.; Correa-Guimaraes, A.; Martín-Gil, J. *Mater. Chem. Phys.* **2011,** *130,* 1094–1102. doi:10.1016/j.matchemphys.2011.08.041
